# Fatty Acid Binding Protein 4 and 5 Play a Crucial Role in Thermogenesis under the Conditions of Fasting and Cold Stress

**DOI:** 10.1371/journal.pone.0090825

**Published:** 2014-03-06

**Authors:** Mas Rizky A. A. Syamsunarno, Tatsuya Iso, Aiko Yamaguchi, Hirofumi Hanaoka, Mirasari Putri, Masaru Obokata, Hiroaki Sunaga, Norimichi Koitabashi, Hiroki Matsui, Kazuhisa Maeda, Keigo Endo, Yoshito Tsushima, Tomoyuki Yokoyama, Masahiko Kurabayashi

**Affiliations:** 1 Department of Medicine and Biological Science, Gunma University Graduate School of Medicine, Gunma, Japan; 2 Education and Research Support Center, Gunma University Graduate School of Medicine, Gunma, Japan; 3 Department of Bioimaging Information Analysis, Gunma University Graduate School of Medicine, Gunma, Japan; 4 Department of Diagnostic Radiology and Nuclear Medicine, Gunma University Graduate School of Medicine, Gunma, Japan; 5 Department of Public Health, Gunma University Graduate School of Medicine, Gunma, Japan; 6 Department of Laboratory Sciences, Gunma University Graduate School of Health Sciences, Gunma, Japan; 7 Department of Molecular Imaging and Radiotherapy, Graduate School of Pharmaceutical Sciences, Chiba University, Chiba, Japan; 8 Department of Complementary and Alternative Medicine, Graduate School of Medicine, Osaka University Hospital, Osaka, Japan; University of Texas Health Science Center at Houston, United States of America

## Abstract

Hypothermia is rapidly induced during cold exposure when thermoregulatory mechanisms, including fatty acid (FA) utilization, are disturbed. FA binding protein 4 (FABP4) and FABP5, which are abundantly expressed in adipose tissues and macrophages, have been identified as key molecules in the pathogenesis of overnutrition-related diseases, such as insulin resistance and atherosclerosis. We have recently shown that FABP4/5 are prominently expressed in capillary endothelial cells in the heart and skeletal muscle and play a crucial role in FA utilization in these tissues. However, the role of FABP4/5 in thermogenesis remains to be determined. In this study, we showed that thermogenesis is severely impaired in mice lacking both FABP4 and FABP5 (DKO mice), as manifested shortly after cold exposure during fasting. In DKO mice, the storage of both triacylglycerol in brown adipose tissue (BAT) and glycogen in skeletal muscle (SkM) was nearly depleted after fasting, and a biodistribution analysis using ^125^I-BMIPP revealed that non-esterified FAs (NEFAs) are not efficiently taken up by BAT despite the robustly elevated levels of serum NEFAs. In addition to the severe hypoglycemia observed in DKO mice during fasting, cold exposure did not induce the uptake of glucose analogue ^18^F-FDG by BAT. These findings strongly suggest that DKO mice exhibit pronounced hypothermia after fasting due to the depletion of energy storage in BAT and SkM and the reduced supply of energy substrates to these tissues. In conclusion, FABP4/5 play an indispensable role in thermogenesis in BAT and SkM. Our study underscores the importance of FABP4/5 for overcoming life-threatening environments, such as cold and starvation.

## Introduction

The endothelium has emerged as a dynamic organ that regulates the systemic lipid metabolism and insulin sensitivity, and as such, endothelial dysfunction contributes to the pathogenesis of overnutrition-related metabolic disorders, such as type 2 diabetes and metabolic syndrome [1], [2]. Endothelial cells express a variety of the genes involved in fatty-acid (FA) metabolism. PPARγ plays a critical role as a master regulator of the transcription of these genes [3], [4], and vascular endothelial growth factor (VEGF)-B secreted from cardiac and skeletal muscle and brown adipose tissue produces fatty acid transport proteins via VEGF receptor 1 and the co-receptor neuropilin 1 in capillary endothelial cells [5], [6]. These molecules promote FA transport from the blood, across endothelial cells, to myocytes and adipocytes, which are highly oxidative cells.

FA-binding proteins (FABPs) are 14- to 15-kDa proteins that bind with high affinity to hydrophobic molecules, such as long-chain FA and eicosanoids, in the cytoplasm, and facilitate the transport of lipids to specific compartments in the cells [7]. Hotamisligil and colleagues have identified the prominent expression of FABP4/5 in adipocytes and macrophages as a critical mediator of the inflammatory and metabolic responses in these cells. Indeed, these researchers demonstrated that the chemical inhibition of the function of FABP4 prevents the insulin resistance and atherosclerosis induced by a high-fat diet in mice [8]. We have recently shown that FABP4/5 are clearly expressed in capillary endothelial cells in the heart and skeletal muscle [9]. Notably, mice doubly deficient for FABP4/5 (DKO mice) exhibit a defective uptake of FA in the heart and red skeletal muscle and a compensatory upregulation of glucose consumption in these tissues during fasting, which implies that capillary endothelial FABP4/5 play a crucial role in supplying FAs to highly oxidative tissues such that these tissues meet their high energy demand [9]. DKO mice further exhibit disturbances of systemic metabolism in response to prolonged fasting, such as elevated levels of serum non-esterified FAs (NEFAs) and marked hypoglycemia [10]. Elevated levels of serum NEFAs occur as a result of impaired uptake of FAs by the heart and skeletal muscle, and pronounced hypoglycemia is induced by the compensatory upregulation of glucose consumption in the heart and skeletal muscle and blunted gluconeogenesis in the liver. Thus, the systemic metabolism in response to fasting is markedly disturbed in DKO mice. However, it remains to be determined whether FABP4/5 are required for thermogenesis under starvation and cold stress.

Brown adipose tissue (BAT) and skeletal muscle play major roles in thermogenesis during cold exposure. BAT is favorably vascularized to permit the dissipation of generated heat and has the extensive sympathetic innervation and abundant mitochondria that are essential for thermogenic activity. Although lipolysis in white adipose tissue (WAT) results in the breakdown of intracellular triacylglycerol (TG) and hence an increased release of NEFAs into the circulation, NEFAs produced by lipolysis within BAT serve dual roles by allosterically activating uncoupling protein 1 (UCP1), a proton-translocating membrane protein, to generate heat instead of ATP synthesis and by undergoing β-oxidation to fuel thermogenesis in the mitochondria. Once activated, brown adipocytes can significantly enhance energy expenditure by combusting intracellular TG stores [11]. The continued activity of BAT in a cold environment further requires the utilization of circulating glucose, NEFAs, and TG-rich lipoproteins (TRLs) [11]–[13]. Glucose uptake may be enhanced by increased expression levels of the glucose transporters Slc2a1 (Glut1) and Slc2a4 (Glut4), and NEFA uptake may be mediated by fatty acid translocase (FAT)/CD36. TRL uptake into BAT is crucially dependent on lipoprotein lipase (LPL) and CD36. Thus, cold exposure induces the thermogenic activity of BAT to maintain euthermia by consuming abundant energy substrates.

Skeletal muscle (SkM) is another crucial tissue for generating heat by shivering and physical activity [14]. Shivering muscles are largely fueled by carbohydrates and lipids. Although the contribution of circulating glucose to total heat generation remains minor (<15% of total heat produced) [15], muscle glycogen becomes the dominant fuel, providing 30–40% of the total heat produced or 75% of the total carbohydrate oxidized. The size of the glycogen reserve causes a large shift in fuel use from carbohydrate dominance to lipid dominance (up to ∼80%) [14]. In addition, there is a significant inverse relationship between the BAT volume and shivering [11], which implies that shivering does contribute to thermogenesis during acute cold exposure. Furthermore, it has been reported that cold exposure increases the basal metabolic rate by 28% after 2 h of exposure [16] and by 80% after 3 h of exposure [11]. Thus, the body temperature is maintained by the non-shivering thermogenesis of BAT and the shivering thermogenesis of SkM through the acute combustion of large amounts of energy substrates, such as TG in BAT, glycogen in SkM, and circulating glucose, NEFAs, and TRLs.

In addition to the disruption of well-known thermoregulatory genes, such as UCP1, PGC1α (PPARγ coactivator 1α), and Dio2 (deiodinase type 2, an enzyme responsible for converting inactive thyroxine T4 to active T3) [17], impaired thermogenesis is also observed in mice deficient for genes associated with FA metabolism [18], including MCAD (medium-chain acyl-CoA dehydrogenase) [19], LCAD (long-chain acyl-CoA dehydrogenase) [20], and DECR (2,4-dienoyl-CoA reductase) [21]. In these knockout mice, hypothermia is apt to appear during prolonged fasting or when energy from lipids is needed. During fasting, these mice also exhibit common metabolic characteristics, such as hypoglycemia, elevated levels of serum NEFAs, and prominent fatty liver [18], [22], which are also observed in DKO mice as described above [10]. Although it should be noted that impaired FA oxidation, decreased shivering and physical activity, and reduced levels of UCP1 and Dio2 mRNA in BAT may cause hypothermia in mice deficient for genes associated with FA metabolism, the precise mechanisms of these processes remain unsolved.

In this study, we showed that thermogenesis is severely impaired in DKO mice shortly after cold exposure only during fasting. Most energy substrates for thermogenesis, such as TG in BAT, glycogen in SkM, and circulating glucose, become unavailable because these are rapidly depleted in DKO mice under a cold environment in the fasted state, resulting in the rapid occurrence of fatal hypothermia.

## Results

### Effects of Cold Stress on Body Temperature

**Figure 1:**
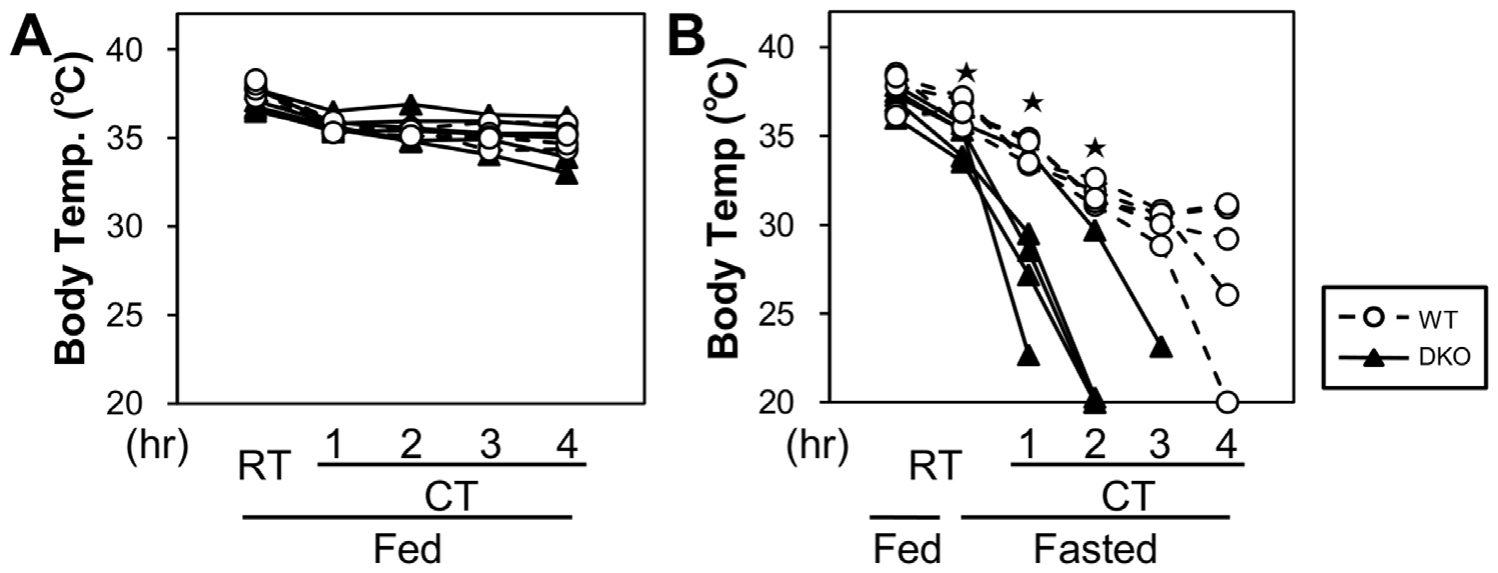
Cold intolerance in FABP4/5 DKO mice with prior fasting. The cold tolerance was tested as described in the methods section. The body temperature was measured from the shaved mid-dorsal body surface. n = 5/group. *p<0.05.

To determine the effects of cold stress, mice were exposed to a cold environment (4°C) for 4 h with or without prior fasting for 20 h (Figure 1). When mice were continuously fed, their body surface temperature was higher than 30°C during cold exposure, and there was no difference in the body surface temperature between wild-type (WT) and DKO mice (Figure 1A). However, the body temperature of all DKO mice that were fasted for 20 h rapidly decreased and dropped to less than 25°C within 3 h (Figure 1B). In contrast, the body temperature of WT mice was higher than 28°C 3 h after fasting. The body temperature of only one WT mouse dropped to less than 25°C 4 h after fasting. The mice in both the fed and fasted states showed similar shivering behavior and similarly reduced physical activity. Thus, the DKO mice exhibited cold intolerance compared with the WT mice when acutely exposed to a cold environment during fasting.

### Effects of Cold Stress on Blood Glucose, Lipids, and Ketone Bodies

**Figure 2:**
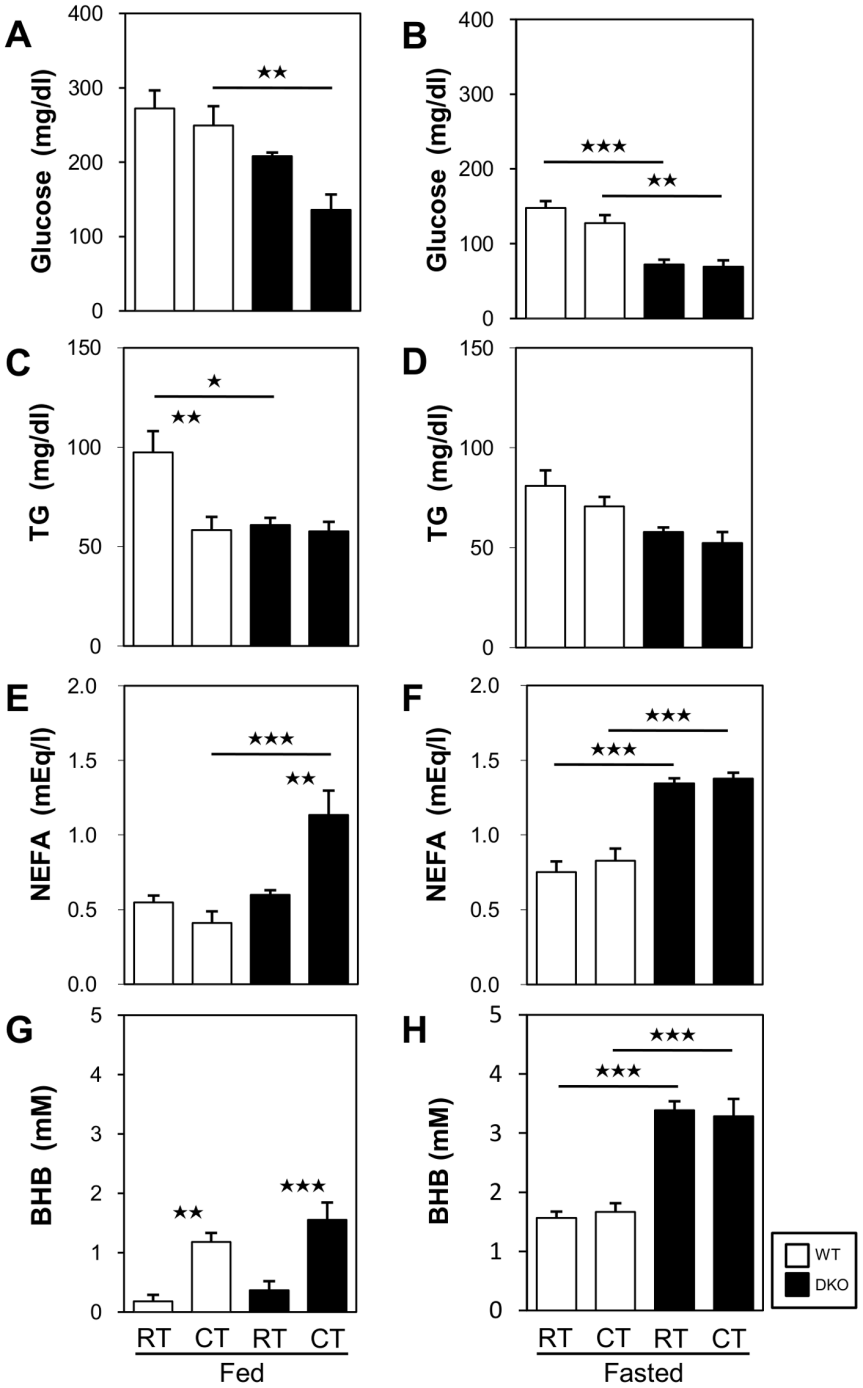
Very low glucose levels in FABP4/5 DKO mice in the fasted state regardless of cold exposure. (A to H) Blood was collected from the retro-orbital plexus before and after cold exposure to measure the serum levels of glucose (A and B), TGs (C and D), NEFAs (E and F), and ketone bodies (G and H) in the fed (A, C, E, and G) and the fasted states (B, D, F, and H). n = 5–6/group. *p<0.05; **p<0.01; ***p<0.001. Note that the blood was collected 4 h after cold exposure for the fed groups and 2 h after cold exposure for the fasted groups because many DKO mice died during prolonged cold exposure in the fasted state.

We then studied some biochemical parameters before and after cold exposure with and without prior fasting. The DKO mice in the fed state presented glucose levels that were decreased after cold exposure and increased NEFAs levels (Figures 2A and 2E), which suggests that these mice exhibit enhanced consumption of glucose and accelerated lipolysis with impaired NEFA uptake by peripheral tissues during cold exposure. The levels of TG and ketone bodies (β-hydroxybutyrate, BHB) were comparable after cold exposure (Figures 2C and 2G). In fasting DKO mice, this lower level of glucose was not altered by cold exposure (Figure 2B). The TG level was not significantly changed by cold exposure (Figure 2D). The levels of NEFAs and ketone bodies were markedly enhanced during fasting [10], and these were not altered by cold exposure (Figures 2F and 2H). Thus, it is noteworthy that the obvious hypoglycemia in DKO mice during fasting was not affected by cold exposure, suggesting that the supply of glucose to the peripheral tissues, including BAT and SkM, is limited during cold exposure in the fasted state.

### Effects of Cold Stress on BAT Weight

**Figure 3:**
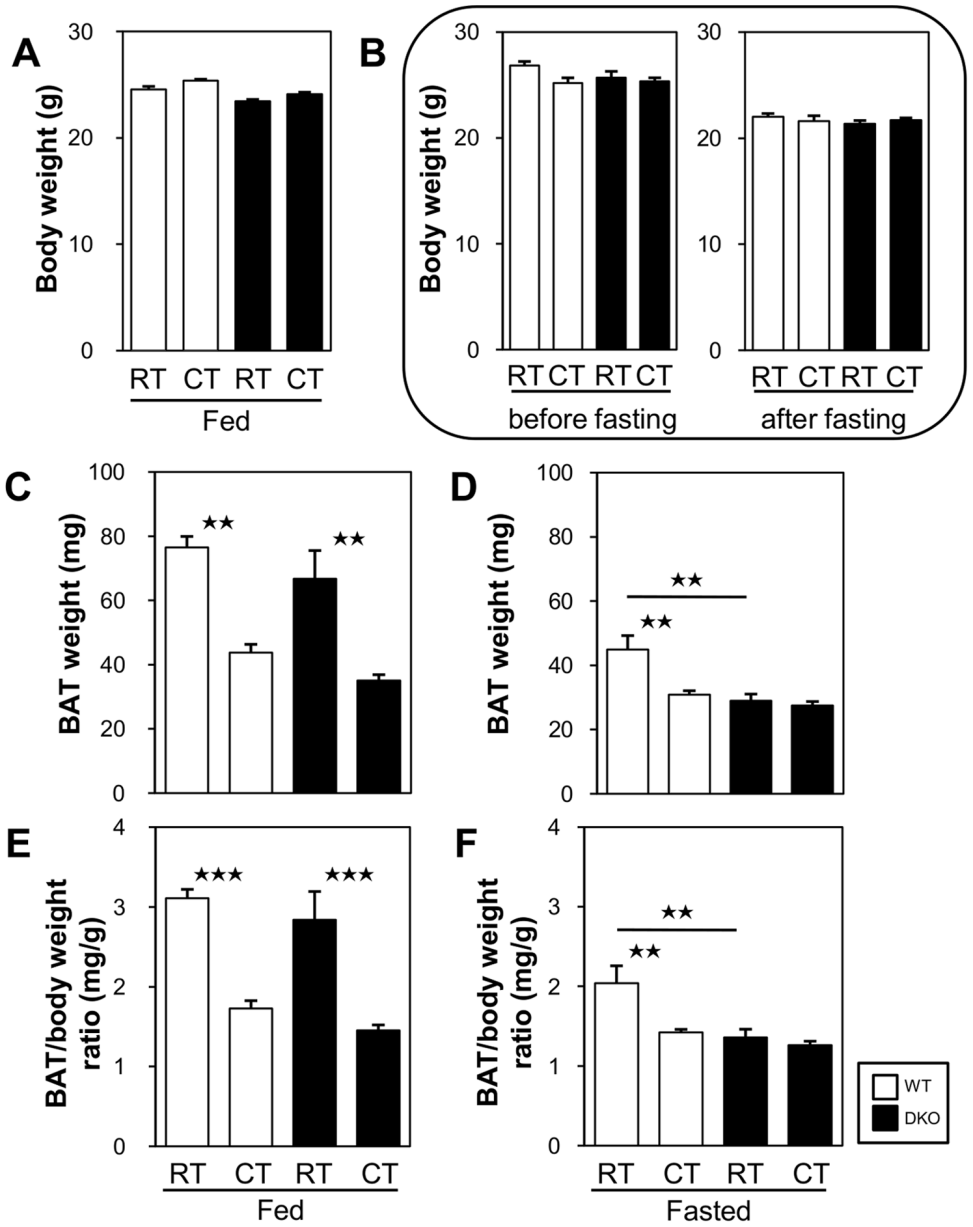
The reduced weight of BAT in the DKO mice subjected to a 20 h fast was not altered after cold exposure. The mice were exposed to a cold environment (4°C) with or without prior fasting. (A and B) Original body weight (BW) of mice in each group before cold exposure. (A) BW of the mice included in Figures 4C and 4E before the experiment. (B) Left panel: BW of mice included in Figures 4D and 4F before fasting. Right panel: BW of mice included in Figures 4D and 4F after a 20 h fast but before cold exposure. (C and D) Weight of interscapular BAT in each group before and after cold exposure in the fed (C) and the fasted states (D). (E and F) The ratio of the BAT weight to the BW before fasting was calculated from the data shown above. Note that the BAT is significantly shrunk after fasting or cold exposure and that the reduced weight of BAT in the DKO mice after a 20 h fast was not altered after cold exposure. n = 4–5/group. *p<0.05; **p<0.01; ***p<0.001.

We then measured the weight of BAT from mice that showed comparable BW with and without fasting (Figures 3A and 3B). In the fed state, the weight of interscapular BAT was significantly reduced after cold exposure in both groups of mice (Figures 3C and 3E). In the fasted state, the weight of BAT before cold exposure was already smaller in the DKO mice than in the WT mice (Figures 3D and 3F), which suggests increased consumption or lipolysis of TG in BAT during fasting. Importantly, the weight of BAT was not decreased even after cold exposure in the DKO mice (Figures 3D and 3F), which strongly suggests that there was inadequate TG storage for thermogenesis in BAT in the DKO mice after fasting.

### Effects of Cold Stress on TG Storage in BAT and Glycogen Storage in SkM

**Figure 4:**
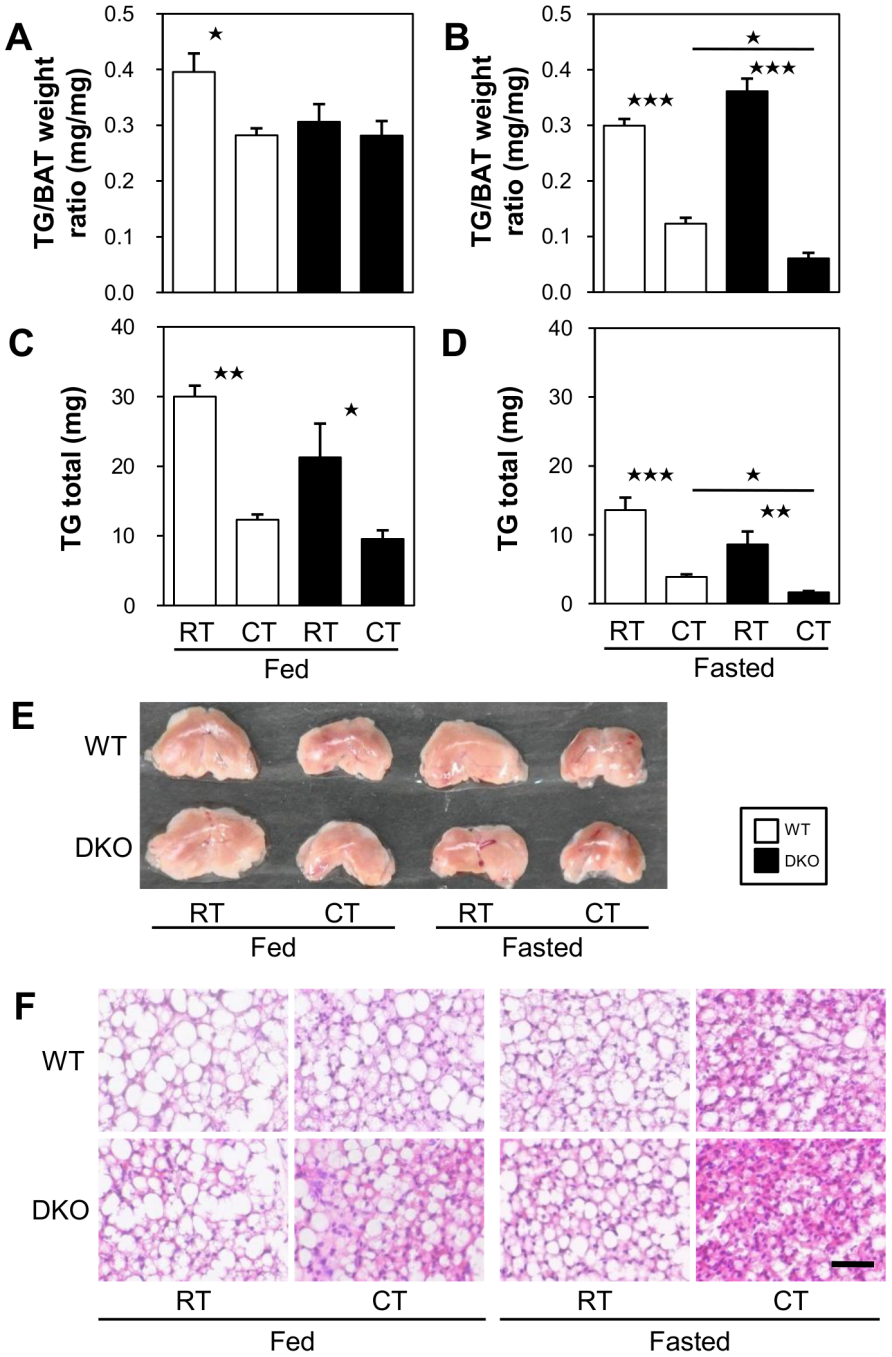
TGs stored in BAT in DKO mice were more depleted after cold exposure in the fasted state. The mice were exposed to a cold environment (4°C) with or without prior fasting. (A and B) TG concentration in BAT. (C and D) The total amount of TGs in interscapular BAT was calculated from the concentration of TGs in BAT and the weight of BAT. n = 4–5/group. *p<0.05; **p<0.01; ***p<0.001. (E) Gross appearance of BAT. (F) Hematoxylin/eosin staining of BAT. Bar indicates 50 µm.

We next examined the TG storage in interscapular BAT. In the fed state, the concentration and total amount of TG in interscapular BAT were not significantly different between the WT and the DKO mice before and after cold exposure (Figures 4A and 4C), suggesting that the amount of TG consumed in BAT is almost equivalent during cold exposure. In the fasted state, however, the concentration and total amount of TG were significantly lower after cold exposure in the DKO mice compared with the WT, suggesting that the available TG storage before fasting becomes nearly empty in the DKO mice after cold exposure (Figures 4B and 4D). Consistent with the TG levels in BAT, the color of BAT was darker, and the multilocular lipid droplets in BAT became smaller (Figures 4E and 4F, respectively).

**Figure 5:**
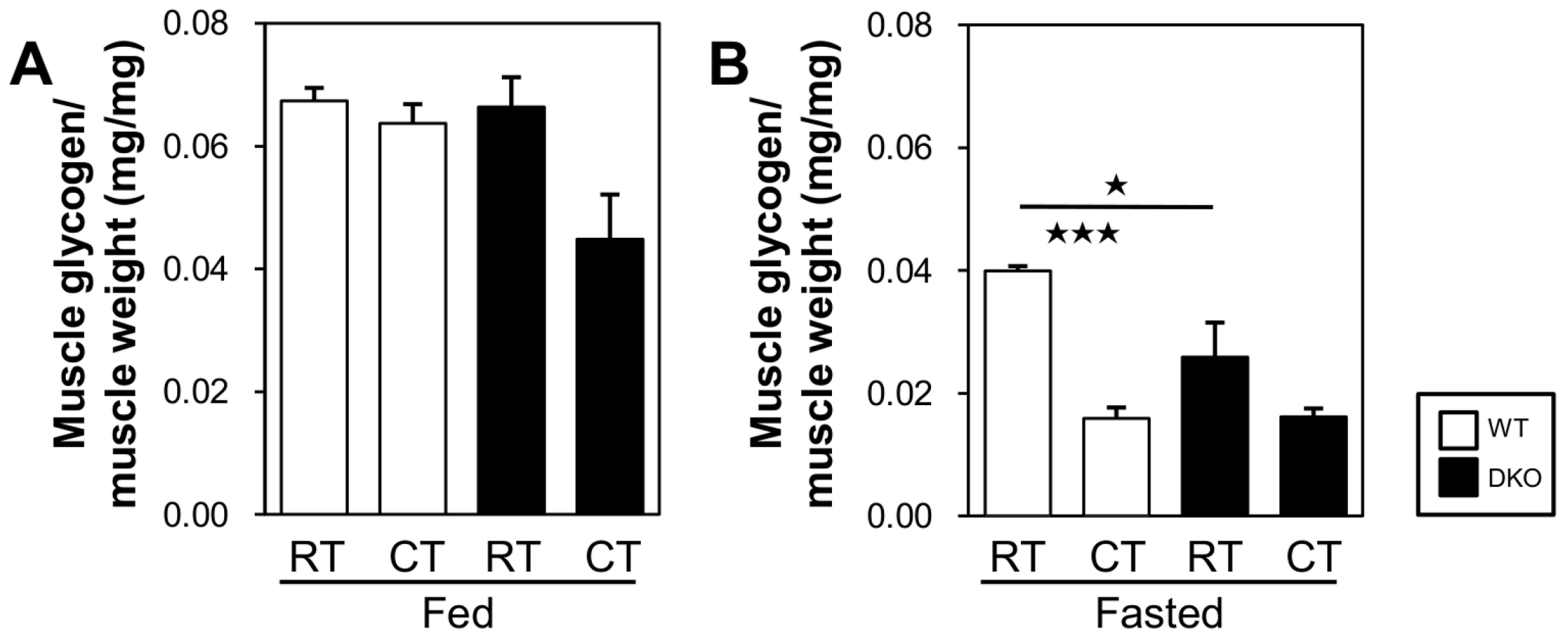
Glycogen storage is depleted in SkM after a 20 h fast. The quadriceps femoris muscle was collected from mice before and after cold exposure (4°C) with or without prior fasting. (A and B) Concentration of glycogen in the quadriceps femoris muscle. n = 4–5/group. *p<0.05; ***p<0.001.

We also examined the glycogen concentration in the quadriceps femoris muscle. In the fed state, the glycogen concentration was marginally reduced in DKO mice after cold exposure (Figure 5A), suggesting that the lower supply of circulating glucose (Figure 2A) causes an increased consumption of stored glycogen for shivering thermogenesis. In the fasted state, the lower level of glycogen concentration in the SkM of the DKO mice was not altered after cold exposure (Figure 5B), suggesting that glycogen storage is exhausted before cold exposure. Thus, as estimated by the TG levels in BAT and the glycogen concentration in SkM, the energy storage was nearly depleted in the DKO mice after a 20 h fast, and this depletion may be one of the major reasons for the lower levels of thermogenic activity observed during cold exposure.

### Effects of Cold Stress on Glucose and FA Uptake in BAT and Skeletal Muscle

**Figure 6:**
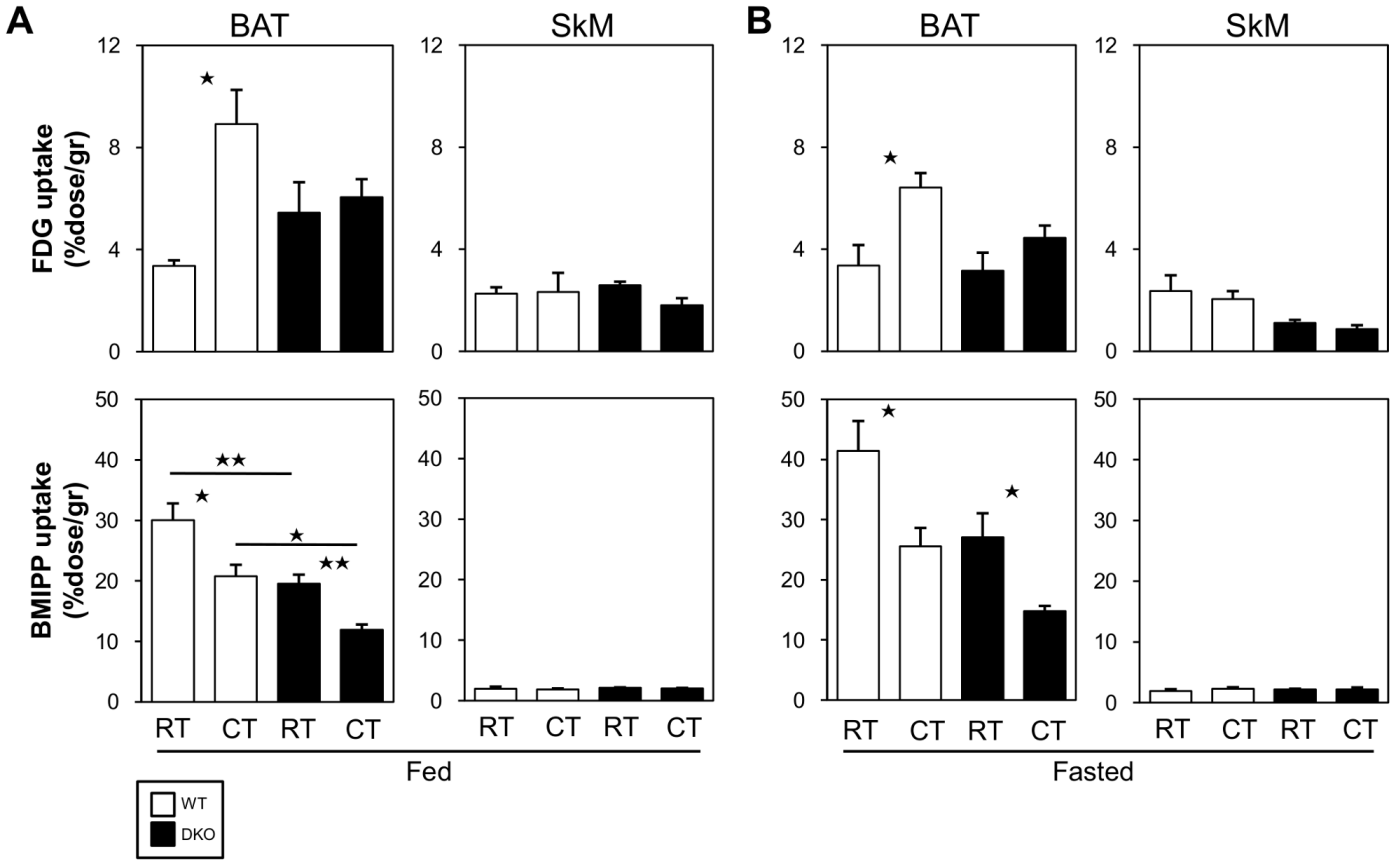
Uptake of ^18^F-FDG and ^125^I-BMIPP by BAT is markedly reduced in DKO mice after cold exposure with prior fasting. (A and B) The mice received intravenous injections of ^18^F-FDG (100 kBq) and ^125^I-BMIPP (5 kBq) via the lateral tail vein before (A) and after a 20 h fast (B). The mice were maintained at room temperature or in cold rooms (4°C) for 2 h and then sacrificed. The uptake of ^18^F-FDG by BAT and the uptake of ^125^I-BMIPP and SkM were counted using a well-type gamma counter (n = 4–6/group). *p<0.05; **p<0.01.

We then estimated the uptake of glucose and NEFAs using the glucose analogue ^18^F-FDG and the FA analogue ^125^I-BMIPP, respectively. In the fed state, the uptake of ^18^F-FDG by BAT in the WT mice was significantly upregulated after cold exposure, whereas the uptake of ^125^I-BMIPP was reduced (Figure 6A). In DKO mice, the uptake of ^18^F-FDG by BAT was not altered after cold exposure, and the uptake of ^125^I-BMIPP was lower than that observed in the WT mice and decreased after cold exposure. In the fasted state, the uptake of ^18^F-FDG was not significantly increased in BAT in DKO mice (Figure 6B). The uptake of ^125^I-BMIPP by BAT was also reduced in the DKO mice after cold exposure (Figure 6B). The uptake of ^18^F-FDG was lower in the SkM of the DKO mice after cold exposure, but this difference was not statistically significant. The uptake of ^125^I-BMIPP by the SkM was very low in both WT and DKO mice and was unaffected by cold exposure. Because the serum level of glucose was markedly reduced in the DKO mice after a fast as well as cold exposure compared with the WT mice (Figures 2A and 2B), the relative uptake of glucose by BAT and SkM was decreased in the DKO mice. Thus, the uptake of energy substrates, such as glucose and NEFAs, into BAT and SkM was severely impaired before and after cold exposure with prior fasting, further supporting the hypothesis that a reduced supply of energy substrates to BAT and SkM could result in impaired thermogenesis.

### Effects of Cold Stress on Expression of FABP3 in BAT of DKO Mice

**Figure 7:**
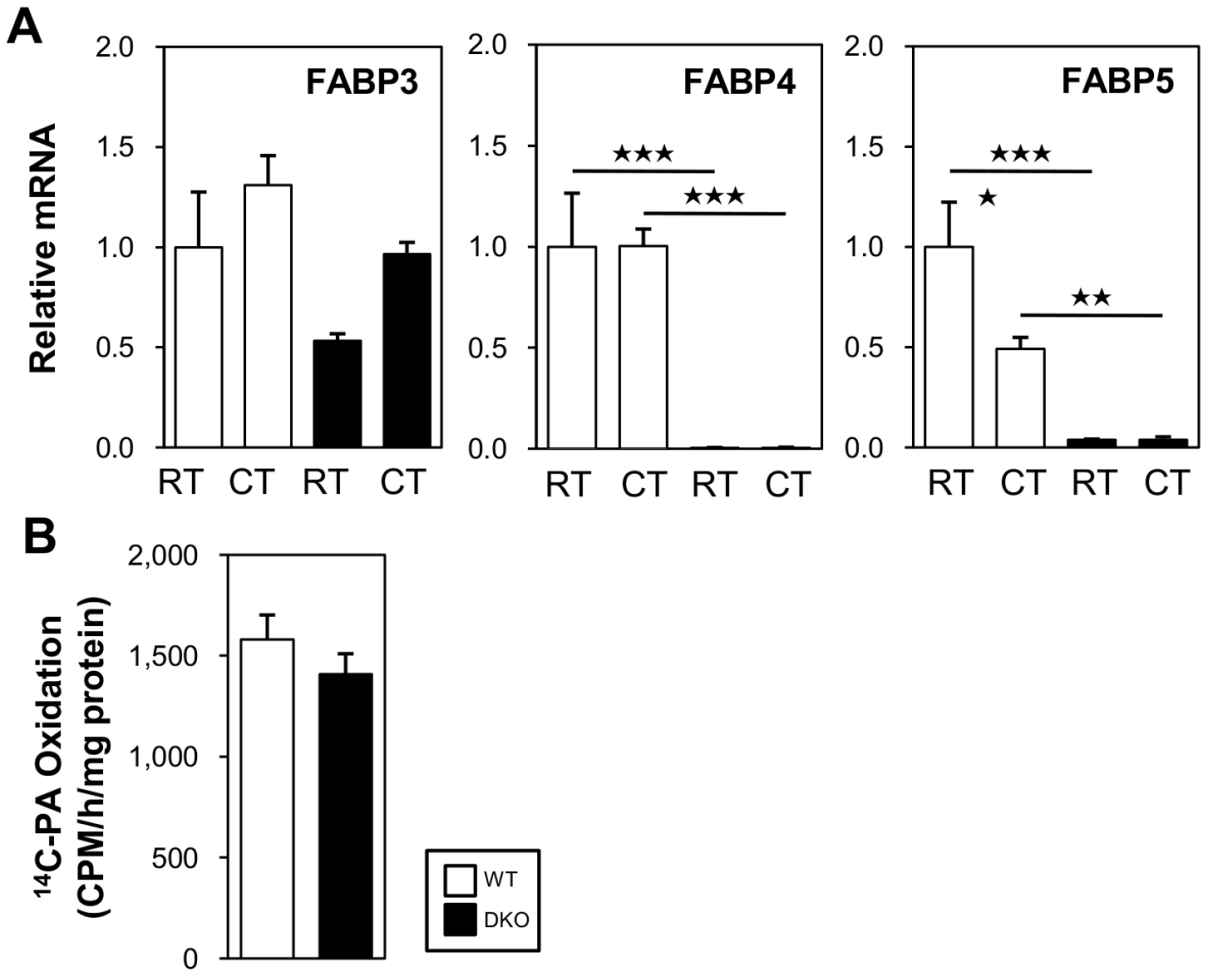
The expression level of FABP3 and FA oxidation capacity were comparable between WT and DKO mice. (A) The mice were maintained at room temperature or in a cold room (4°C) for 4 h in the fed state. The total RNA from BAT was extracted for quantitative real-time PCR. n = 4–5/group. (B) The FA oxidation by BAT homogenates was estimated *in vitro*. The mice were placed in a cold room (4°C) for 2 h and then sacrificed. n = 4–5/group. *p<0.05; **p<0.01; ***p<0.001.

The expression of FABP3, a member of the FABP family, is induced in BAT by cold stress and is essential for efficient FA oxidation in BAT and for cold tolerance [23]–[25]. We studied the expression of FABP3, FABP4, and FABP5 in the BAT of WT and FABP4/5 DKO mice with and without cold exposure (Figure 7A). In the WT mice, the expression of FABP3 was marginally induced by cold stress. However, the expression of FABP4 was not altered, and the expression of FABP5 was reduced. In the DKO mice, the basal expression of FABP3 tended to be lower than that found in the WT mice, and the expression of this protein was slightly (but not significantly) induced by cold exposure. The expression levels of FABP4/5 were negligible. This expression pattern suggests that FABP4/5 and FABP3 do not have redundant functions in thermogenesis in response to cold stress. In addition, it is noteworthy that BAT homogenates prepared from WT and DKO mice equally oxidized exogenously supplied palmitate (Figure 7B), whereas FA oxidation is impaired in FABP3-deficient brown adipocytes [23].

### Effects of Cold Stress on the Expression of the Genes Required for Thermogenesis

**Figure 8:**
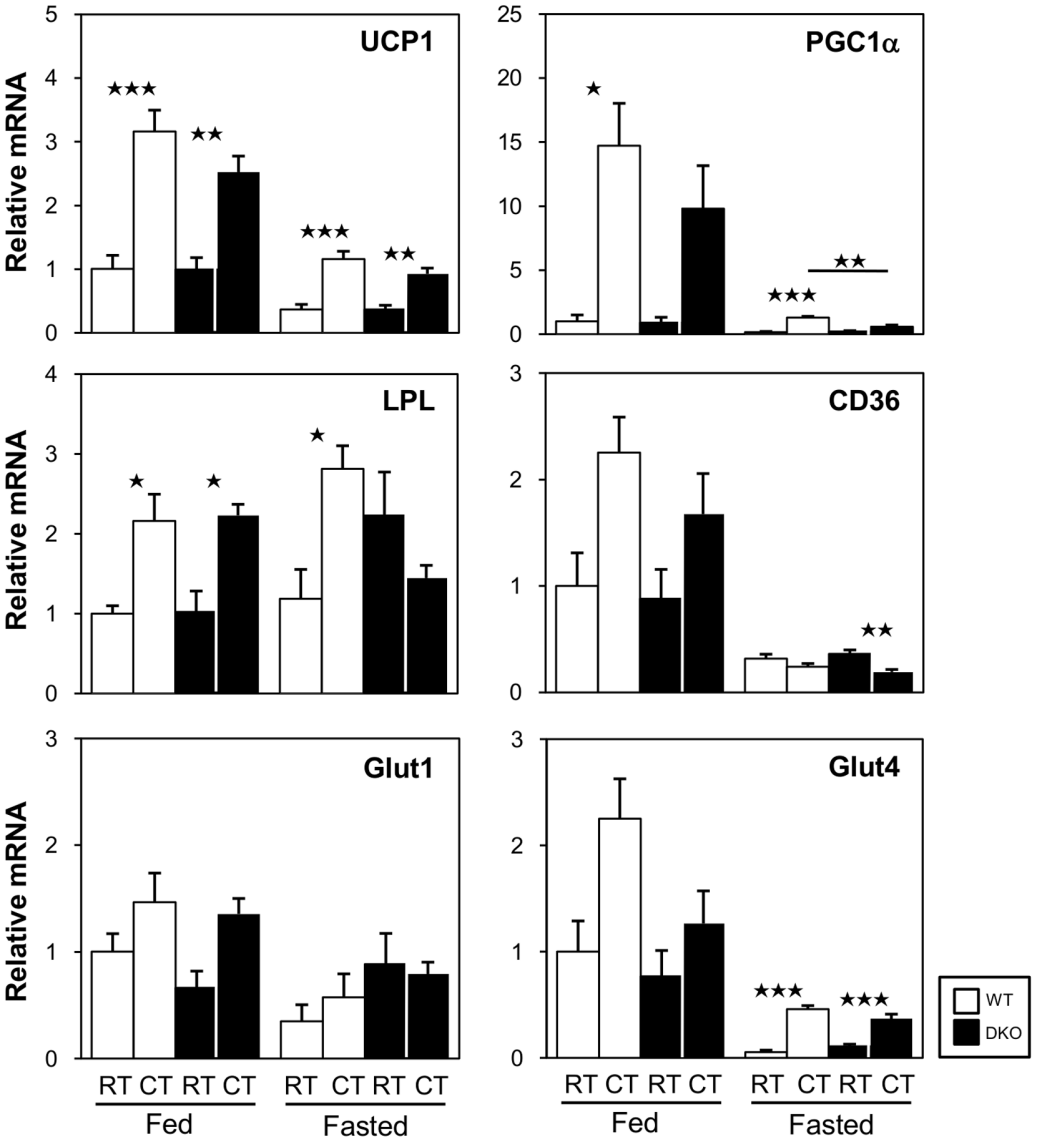
Reduced induction of genes associated with thermogenesis in BAT in both WT and DKO mice after cold exposure in the fasted state. The mice were maintained at room temperature or in a cold room (4°C) for 4 h with or without prior fasting. The total RNA from BAT was extracted for quantitative real-time PCR. n = 4–5/group. *p<0.05; **p<0.01; ***p<0.001.

We next evaluated the expression levels of genes associated with thermogenesis in BAT in response to cold exposure (Figure 8). In the fed state, the mRNA expression of UCP1, PGC1α, and LPL was upregulated by cold exposure. In the fasted state, the expression of all transcripts with the exception of LPL was markedly reduced before cold exposure in both WT and DKO mice. Although the expression of UCP1 and glut4 mRNA was upregulated by cold exposure, the induction level was similar to or less than their basal expression in the fed state before cold exposure. The expression of LPL mRNA was not induced by cold exposure in DKO mice. The expression of CD36 was very low in both WT and DKO mice and was significantly reduced in DKO mice after cold exposure. In summary, the expression levels of genes relevant to thermogenesis were low after fasting in both WT and DKO mice, suggesting that the thermogenic capacity of BAT is significantly reduced at the mRNA level during fasting in general. The lack of induction of LPL and the reduced expression of CD36 further suggest that the uptake of circulating NEFAs and TRLs and the lipolysis of TGs in TRLs may be impaired in DKO mice after cold exposure in the fasted state.

## Discussion

The present study provides several lines of evidence indicating that FABP4/5 play a crucial role in thermogenesis, which is required for the maintenance of the body temperature under cold stress. First, the body temperature of the DKO mice was rapidly reduced after cold exposure in the fasted state. Second, the serum level of glucose was extremely low in the DKO mice in the fasted state regardless of cold exposure. Third, the TG levels in BAT and glycogen concentration in SkM were very low in the DKO mice after fasting, even before cold exposure. Fourth, the uptake of both ^18^F-FDG and ^125^I-BMIPP by BAT and SkM was not enhanced in the DKO mice during cold exposure after fasting. Heat production during acute cold exposure is mainly regulated both by non-shivering thermogenesis by BAT and by shivering thermogenesis by SkM. In addition, there is a significant inverse relationship between the BAT volume and shivering [11], implying that the two thermoregulatory mechanisms support each other. Therefore, it is plausible that heat is no longer produced if there is no fuel, and it is not supplemented by the two heat-generating tissues. Taken together, the rapid hypothermia observed in the DKO mice after fasting is most likely caused by the depletion of the energy storage in BAT and SkM and the lower supply of energy substrates to these tissues (refer to Figure S1 in file S1 for further details). Thus, this study provides the first evidence that thermogenesis is impaired in mice with defective FA transport via capillary endothelial cells from the circulation to fat-burning tissues, i.e., in mice in which that systemic metabolism in response to fasting is markedly disturbed.

We excluded the possibility that the activation of the sympathetic nervous system is impaired by showing that lipolysis, as estimated by a reduction in TG storage in BAT and increased serum levels of NEFAs, was promoted by fasting as well as by cold exposure. We further confirmed that FA oxidation by BAT homogenates was comparable, implying that FABP4/5 per se are not involved in FA oxidation in BAT. The expression level of FABP3, a thermoregulatory FABP, was not different between WT and DKO mice. Furthermore, the results of a series of real-time PCR analyses indicate that the expression of thermoregulatory genes, such as UCP1 and PGC1α, was similarly reduced in both WT and DKO mice during fasting with cold exposure. Collectively, our findings suggest that severe hypothermia is unlikely to be due to impaired activation of the sympathetic nerve system, disturbed FA oxidation, or the excessive repression of the thermogenic gene program of brown fat in DKO mice.

We found remarkable differences between WT and DKO mice in the TG levels in BAT, the glycogen levels in SkM, and the uptake of circulating energy substrates only during fasting. Importantly, we extensively estimated the storage and the supply of energy substrates for both BAT and SkM before and after fasting with or without cold exposure in a single study. FA-metabolism-deficient mice, such as KO mice for the MCAD, LCAD, and DECR genes [19]–[21], [26], exhibit metabolic characteristics very similar to those found in the FABP4/5 DKO mice during prolonged fasting (e.g., hypoglycemia, elevated levels of serum NEFAs, and fatty liver). Cold intolerance is another typical finding in these mice. However, the mechanism of hypothermia has not been well clarified. Although it has been reported that impaired FA oxidation, reduced shivering and physical activity, and decreased expression of UCP1 and Dio2 may cause hypothermia in these mice, the contribution of energy substrates to both BAT and SkM before and after fasting with or without cold exposure has yet to be evaluated. It is worth investigating whether energy depletion in both BAT and SkM after fasting is a common mechanism underlying cold-induced hypothermia in mice with defective FA utilization.

In summary, appropriate re-distribution of energy substrates fails to occur in DKO mice during fasting, resulting in a shortage of energy stores for thermogenesis, such as glycogen in SkM and TGs in BAT, and a reduced supply of energy substrates to these tissues. Without sufficient fuel, DKO mice exhibit rapid hypothermia in a cold environment during fasting. Our data provide the first proof that FABP4/5 play an indispensable role in thermogenesis during fasting, which underscores the importance of FABP4/5 for overcoming life-threatening environments, such as cold and starvation.

## Materials and Methods

### Mice and Sample Collection

Mice with homozygous null mutations in *Fabp4* (*Fabp4*−/−) or *Fabp5* (*Fabp5*−/−) were generated as described elsewhere [27], [28]. Mice doubly deficient for *Fabp4* and *Fabp5* (*Fabp4/5* DKO) were generated from an intercross between *Fabp4* (−/−) and *Fabp5* (−/−) mice, as described previously [29]. Control male wild-type C57BL6j mice were purchased from Japan SLC, Inc. Their age was almost the same as that of the FABP4/5 DKO mice, and their body weights were comparable at the date of the experiments (Figures 3A and 3B). The Institutional Animal Care and Use Committee (Gunma University Graduate School of Medicine) approved all of the studies. The mice were housed in a temperature-controlled room (22°C) with a 12 h light/12 h dark cycle and were given unrestricted access to water and standard chow. Twelve-week-old male mice were used. For the fasting experiments, the mice were housed individually, and food was withdrawn for 20 h; water was provided *ad libitum*. One sample of BAT and one sample of SkM were snap-frozen in liquid nitrogen and maintained at −80°C until use, and another sample of each was preserved for histological examination.

### Cold Tolerance Test

The cold tolerance was tested by exposing individually housed fasted (20 h) or non-fasted mice to 4°C for a maximum of 4 h or until their body temperature dropped to less than 25°C [21]. It has been shown that mice with body temperatures below 25°C do not recover, and thus this body temperature can be considered terminal without using death as an end-point [30]. The body temperature was measured from the shaved mid-dorsal body surface using a ThermoScan thermometer (PRO 4000, Braun, Kronberg, Germany) as described previously [21]. Blood was collected from the retro-orbital plexus before and after cold exposure to measure the biochemical parameters.

### Measurement of Blood Parameters

The blood glucose was measured with a Glutest sensor (Sanwa Kagaku, Aichi, Japan). The serum levels of triglycerides (Triglyceride E-test, Wako Chemical, Osaka), non-esterified fatty acids (NEFA C-test, Wako Chemical, Osaka), and ketone bodies (EnzyChrom Ketone Body Assay Kit, Bio Assay Systems, CA, USA) were measured according to the manufacturers’ protocols.

### Triglyceride Measurement in BAT

The BAT samples were homogenized with RIPA buffer (50 mM Tris-HCl, pH 7.4, 1% NP40, 0.25% Na-deoxycholate, 150 mM NaCl, and 1 mM EDTA) and centrifuged at 18,000×*g* and 4°C for 10 min. The lipids in the supernatants were extracted with methanol/chloroform (1∶2), evaporated with NO_2_, and dissolved in isopropanol. The triglyceride levels were determined using the Wako Triglyceride E-test (Wako Chemical, Osaka).

### Glycogen Measurement in SkM

The SkM was powder-pulverized in liquid nitrogen, and a 10 mg sample was homogenized in distilled water and boiled at 99°C. After centrifugation, the supernatants were collected to measure the glycogen concentration (BioVision).

### Histological Analysis

The liver samples were fixed with 4% paraformaldehyde and embedded in paraffin. The BAT was stained with hematoxylin and eosin.

### RNA Isolation and Reverse Transcription (RT)–PCR

The total RNA from various organs was isolated using the RNAiso Plus reagent (Takara, Japan). Semi-quantitative RT-PCR was performed with an RT-PCR kit (Takara, Japan) according to the manufacturer’s protocol. Quantitative real-time PCR was performed using the SYBR Green PCR Master Mix (Applied Biosystems) according to the manufacturer’s instructions. The expression level of the target gene was normalized to the mRNA level of TATA-binding protein (TBP). The gene-specific primers for cDNA were described previously [31]–[34] or are listed in Table S1 in file S1.

### Biodistribution of ^125^I-BMIPP (15-(p-iodophenyl)-3-(R,S)-methyl Pentadecanoic Acid) and ^18^F-FDG (2-fluorodeoxyglucose)

The biodistribution of ^125^I-BMIPP and ^18^F-FDG was determined as described previously [3]. The mice received intravenous injections of ^125^I-BMIPP (5 kBq) and ^18^F-FDG (100 kBq) via the lateral tail vein in a total volume of 100 µl. ^125^I-BMIPP was a gift from Nihon Medi-Physics Co. Ltd., and ^18^F-FDG was obtained from batches prepared for clinical PET imaging at Gunma University. The animals were sacrificed 2 h after injection. The isolated tissues were weighed and counted using a well-type gamma counter (ARC-7001, ALOKA). Each experiment was performed at least twice.

### FA Oxidation in BAT

The β-oxidation ability of BAT homogenates was examined as previously described [35]. Twenty milligrams of BAT were manually homogenized using a Potter-Elvehjem homogenizer in 400 µl of ice-cold buffer A (100 mM KCl, 40 mM Tris-HCl, 10 mM Tris base, 5 mM MgCl_2_-6H_2_O, 1 mM EDTA, and 1 mM ATP [pH 7.4]). The lysate was centrifuged at 420×*g* and 4°C for 5 min, and 40 µl of the supernatant was added to 160 µl of the reaction mixture (100 mM sucrose, 10 mM Tris-HCl, 5 mM KH_2_PO_4_, 1 mM MgCl_2_, 2 mM L-carnitine, 0.1 mM malate, 2 mM ATP, 0.05 mM coenzyme A, 1 mM dithiothreitol, 0.2 mM EDTA, and 0.2 µCi ^14^C-palmitic acid [Perkin Elmer, CT, USA] complexed to fatty-acid-free albumin at a 3∶1 molar ratio [pH 7.4]). The reactions were performed in an Eppendorf tube and were placed in an incubator at 37°C for 60 min. The mixture was transferred to a new Eppendorf tube containing 200 µl of 6 N HCl. Filter paper containing 20 µl of ethanolamine was previously placed in the tube cap. The sample was further incubated at room temperature for 1 h to collect the ^14^CO_2_ trapped in the filter paper. The radioactivity in ^14^CO_2_ was quantified using liquid scintillation counting. The value was normalized with the lysate protein concentration.

### Statistical Analysis

The statistical analysis was performed using unpaired Student’s t-test for two samples. One-way ANOVA was performed for more than three samples, and Bonferroni’s post-hoc multiple comparison tests were performed to evaluate the differences between the control and experimental groups. A p-value of less than 0.05 was considered statistically significant. The data are presented as the means ± S.E.

## Supporting Information

File S1 **Figure S1, Working model of rapid hypothermia of DKO mice under a cold environment in the fasted state.** In the fed state, the energy storage, such as TGs in BAT and glycogen in SkM, is adequate against acute exposure to a cold environment in both WT and DKO mice, although the serum glucose is slightly decreased shortly after cold exposure and the NEFA uptake by BAT is inefficient in DKO mice. The expression levels of thermoregulatory genes are almost comparable between these mice. In the fasted state, TG storage in BAT and glycogen storage in SkM are markedly reduced. In WT mice, however, there are higher levels of remaining TGs and glycogen compared with those found in DKO mice. In addition, the serum level of glucose is maintained by gluconeogenesis and a lower consumption by peripheral tissues (a shift in fuel use to lipid dominance). Circulating NEFAs can be taken up by BAT. The glucose uptake by BAT is also enhanced by cold exposure. These forms of energy storage and supply in BAT and SkM are adequate for thermogenesis during short durations of cold exposure. In DKO mice, however, the storage of both TGs in BAT and glycogen in SkM are nearly depleted after a 20-h fast. The serum level of glucose is markedly reduced by accelerated consumption of glucose in the heart and red skeletal muscle as well as by blunted gluconeogenesis after a fast [9], [10]. Although the serum levels of NEFAs are markedly increased, NEFA is not efficiently taken up by BAT. A lower uptake of glucose and NEFA by BAT appears to accelerate the rapid disappearance of TGs. The amount of glucose taken up by SkM is lower, as estimated by the serum glucose level and ^18^F-FDG uptake, which may facilitate the disappearance of glycogen. The expression level of thermoregulatory genes is low in the fasted state in both WT and DKO mice, and their induction by cold exposure is limited in both. The lower mRNA expression levels of LPL and CD36 after cold exposure may cause a diminished uptake of TRLs by BAT in DKO mice. Thus, most energy substrates for thermogenesis, such as TG in BAT, glycogen in SkM, and circulating glucose, become unavailable because they are rapidly depleted in DKO mice in the fasted state during cold exposure, resulting in the rapid occurrence of fatal hypothermia. The sizes of the circles, arrows, and words indicate the relative amounts of energy storage, influx, and gene expression, respectively. TRL; TG-rich lipoprotein. **Table S1, Primers for Real-Time PCR.**
(DOCX)
