# Supplementary material for: Fatty Acid Binding Protein 4 and 5 Play a Crucial Role in Thermogenesis under the Conditions of Fasting and Cold Stress
Source: PLoS One. 2014 Mar 6;9(3):e90825. doi: 10.1371/journal.pone.0090825 (PMC3946242; doi:10.1371/journal.pone.0090825)
Supplement: File S1 — Figure S1, Working model of rapid hypothermia of DKO mice under a cold environment in the fasted state. In the fed state, the energy storage, such as TGs in BAT and glycogen in SkM, is adequate against acute exposure to a cold environment in both WT and DKO mice, although the serum glucose is slightly decreased shortly after cold exposure and the NEFA uptake by BAT is inefficient in DKO mice. The expression levels of thermoregulatory genes are almost comparable between these mice. In the fasted state, TG storage in BAT and glycogen storage in SkM are markedly reduced. In WT mice, however, there are higher levels of remaining TGs and glycogen compared with those found in DKO mice. In addition, the serum level of glucose is maintained by gluconeogenesis and a lower consumption by peripheral tissues (a shift in fuel use to lipid dominance). Circulating NEFAs can be taken up by BAT. The glucose uptake by BAT is also enhanced by cold exposure. These forms of energy storage and supply in BAT and SkM are adequate for thermogenesis during short durations of cold exposure. In DKO mice, however, the storage of both TGs in BAT and glycogen in SkM are nearly depleted after a 20-h fast. The serum level of glucose is markedly reduced by accelerated consumption of glucose in the heart and red skeletal muscle as well as by blunted gluconeogenesis after a fast [9], [10]. Although the serum levels of NEFAs are markedly increased, NEFA is not efficiently taken up by BAT. A lower uptake of glucose and NEFA by BAT appears to accelerate the rapid disappearance of TGs. The amount of glucose taken up by SkM is lower, as estimated by the serum glucose level and 18F-FDG uptake, which may facilitate the disappearance of glycogen. The expression level of thermoregulatory genes is low in the fasted state in both WT and DKO mice, and their induction by cold exposure is limited in both. The lower mRNA expression levels of LPL and CD36 after cold exposure may cause a diminished uptake of TRLs by B [file pone.0090825.s001.docx]

**Figure S1. Working model of rapid hypothermia of DKO mice under a cold environment in the fasted state.**


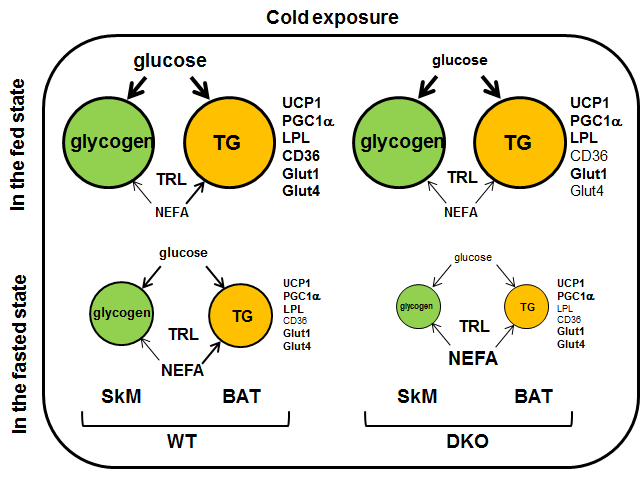


In the fed state, the energy storage, such as TGs in BAT and glycogen in SkM, is adequate against acute exposure to a cold environment in both WT and DKO mice, although the serum glucose is slightly decreased shortly after cold exposure and the NEFA uptake by BAT is inefficient in DKO mice. The expression levels of thermoregulatory genes are almost comparable between these mice. In the fasted state, TG storage in BAT and glycogen storage in SkM are markedly reduced. In WT mice, however, there are higher levels of remaining TGs and glycogen compared with those found in DKO mice. In addition, the serum level of glucose is maintained by gluconeogenesis and a lower consumption by peripheral tissues (a shift in fuel use to lipid dominance). Circulating NEFAs can be taken up by BAT. The glucose uptake by BAT is also enhanced by cold exposure. These forms of energy storage and supply in BAT and SkM are adequate for thermogenesis during short durations of cold exposure. In DKO mice, however, the storage of both TGs in BAT and glycogen in SkM are nearly depleted after a 20-h fast. The serum level of glucose is markedly reduced by accelerated consumption of glucose in the heart and red skeletal muscle as well as by blunted gluconeogenesis after a fast [[9](#_ENREF_9),[10](#_ENREF_10)]. Although the serum levels of NEFAs are markedly increased, NEFA is not efficiently taken up by BAT. A lower uptake of glucose and NEFA by BAT appears to accelerate the rapid disappearance of TGs. The amount of glucose taken up by SkM is lower, as estimated by the serum glucose level and ^18^F-FDG uptake, which may facilitate the disappearance of glycogen. The expression level of thermoregulatory genes is low in the fasted state in both WT and DKO mice, and their induction by cold exposure is limited in both. The lower mRNA expression levels of LPL and CD36 after cold exposure may cause a diminished uptake of TRLs by BAT in DKO mice. Thus, most energy substrates for thermogenesis, such as TG in BAT, glycogen in SkM, and circulating glucose, become unavailable because they are rapidly depleted in DKO mice in the fasted state during cold exposure, resulting in the rapid occurrence of fatal hypothermia. The sizes of the circles, arrows, and words indicate the relative amounts of energy storage, influx, and gene expression, respectively. TRL; TG-rich lipoprotein.

**Table S1. Primers for Real-Time PCR**

| **Primer** | **Forward** | **Reverse** |
| --- | --- | --- |
| FABP3 | catcgagaagaacggggata | tgccatgagtgagagtcagg |
| FABP4 | aagaagtgggagtgggcttt | tcgactttccatcccacttc |
| FABP5 | caaaaccgagagcacagtga | tttgaccgctcactgaattg |
| FAT/CD36 | GAGCAACTGGTGGATGGTTT | TGGGTTTTGCACATCAAAGA |
| Glut1 | TCAACACGGCCTTCACTG | CACGATGCTCAGATAGGACATC |
| Glut4 | ACTCTTGCCACACAGGCTCT | CCTTGCCCTGTCAGGTATGT |
| LPL | TCTGTACGGCACAGTGG | CCTCTCGATGACGAAGC |
| TBP | ACCCTTCACCAATGACTCCTATG | TGACTGCAGCAAATCGCTTGG |
| UCP1 | CTGCCAGGACAGTACCCAAG | TCAGCTGTTCAAAGCACACA |

FABP, fatty acid binding protein; FAT, fatty acid translocase; Glut, glucose transporter; LPL, lipoprotein lipase; TBP, TATA binding protein; UCP1, uncoupling protein 1
